# Chemotherapy‐induced leukopenic septic shock treated with veno‐arterial extracorporeal membrane oxygenation: A case report

**DOI:** 10.1002/ccr3.6979

**Published:** 2023-02-23

**Authors:** Atsumi Hoshino, Shingo Ichiba, Junya Ishikawa, Yusuke Seino, Takuo Yoshida, Nobuo Sato, Takeshi Nomura

**Affiliations:** ^1^ Department of Intensive Care Medicine Tokyo Women's Medical University Tokyo Japan

**Keywords:** extracorporeal membrane oxygenation, management, neutropenia, septic shock

## Abstract

We present a case of chemotherapy‐induced leukopenic septic shock treated with veno‐arterial extracorporeal membrane oxygenation (VA‐ECMO). Although the indication for VA‐ECMO for septic shock in immunosuppressed states remains controversial, her relatively young age and a slightly increasing leukocyte count led to VA‐ECMO induction and resulted in recovery.

## Graphical abstract

A woman in her 40s was admitted with leukopenic sepsis after chemotherapy for an invasive mole. Despite the conventional intensive treatment, her condition deteriorated on ICU day 3. Induction of VA‐ECMO supported her circulation and resulted in her enduring septic shock. VA‐ECMO support for leukopenic septic shock could be useful in relatively young patients with a manageable underlying disease.

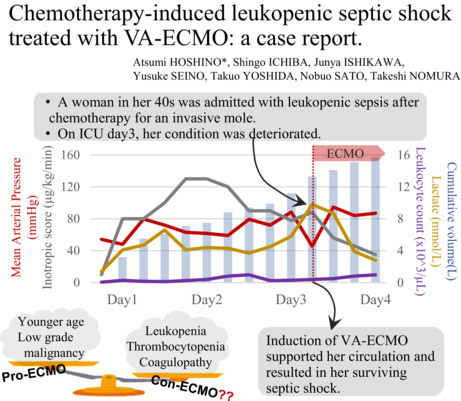

## 1 INTRODUCTION

Recent guidelines have listed sepsis‐induced cardiomyopathy as an emerging indication^1^ for VA‐ECMO, although its use in refractory septic shock remains controversial. Moreover, the efficacy of VA‐ECMO for immunocompromised patients with septic shock is unknown. Herein, we report a case of leukopenic septic shock treated with VA‐ECMO. Written informed consent was obtained from the patient for the publication of this report.

## 2 CASE HISTORY/EXAMINATION

A woman in her 40s underwent chemotherapy (methotrexate, etoposide, and actinomycin D) for an invasive mole. Six days later, she presented with fever, stomatitis, and erythema. On the tenth day, her leukocyte count was 80/μL. She was admitted with a diagnosis of febrile neutropenia due to pneumonia or stomatitis. Cefepime and acyclovir were initiated for the infection and a granulocyte‐stimulating factor for neutropenia.

On day 2 of admission, she developed septic shock and was admitted to the intensive care unit (ICU); ventilatory management, fluid loading, and vasopressors were initiated. Blood tests showed marked leukopenia and thrombocytopenia. Gram‐positive cocci and gram‐negative rods were detected in the blood cultures; hence, cefepime was escalated to meropenem and vancomycin. Echocardiography showed hypercontraction of the left ventricle.

**FIGURE 1:**
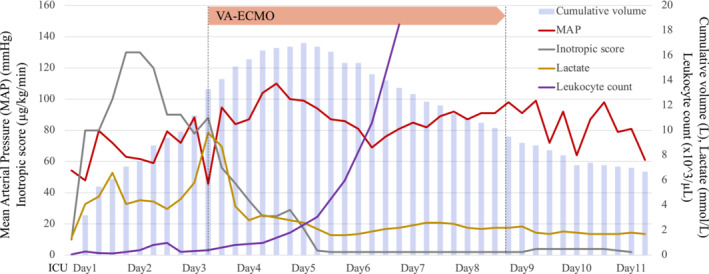
Clinical course before and after veno‐arterial extracorporeal membrane oxygenation (ECMO) support. On day 3 of intensive care unit admission, the mean arterial pressure dropped rapidly and lactate levels elevated, despite fluid resuscitation and massive inotropic support. After ECMO induction, the mean arterial pressure stabilized and lactate levels decreased rapidly. Leukocyte count increased to 5000/μL on day 4 of ECMO support.

On day 2 of ICU admission, serum lactate reached peak levels with a subsequent decline; however, fluid loads and vasopressors could not be reduced to maintain the mean arterial pressure. The cumulative fluid balance reached +9 L, and the inotropic score increased to 130 μg/kg/min (inotropic score = dobutamine dose [μg/kg/min] + {epinephrine dose [μg/kg/min] + norepinephrine dose [μg/kg/min]} × 100) (Figure 1). Echocardiography revealed left ventricular hypokinesis indicative of reverse Takotsubo cardiomyopathy. Coagulopathy, elevated levels of liver enzymes and decreased renal function were observed. Renal replacement therapy was initiated due to oliguria. On day 3 of ICU admission, the leukocyte count increased to 400/μL; however, lactate levels increased to 9.8 mmol/L and the mean arterial pressure decreased rapidly to 40 mmHg. At this point, the cumulative volume amounted to +14 L, the inotropic score was 88 μg/kg/min, and echocardiography revealed a left ventricular ejection fraction of less than 20%. Conventional management was no longer viable, therefore, VA‐ECMO was introduced at an initial flow of 2.3 L/min.

Thereafter, the patient's circulation stabilized. Vasopressors, except for a reduced dose of dobutamine, were tapered and terminated on day 3 of VA‐ECMO support. Her leukocyte count exceeded 5000/μL on day 4. Left ventricular hypokinesis gradually recovered, and she was weaned off VA‐ECMO on day 7. Although she developed empyema, her respiratory function improved; therefore, she was weaned off the ventilator on day 16 of ICU admission. She was transferred to the general ward on day 29 of ICU admission and later to a rehabilitation hospital on day 64 of hospital admission.

## 3 DISCUSSION

The role of VA‐ECMO in adult septic shock has not been fully established. Although the Extracorporeal Life Support Organization (ELSO) interim guidelines in 2021 list septic shock as one of the emerging indications for VA‐ECMO,^1^ Surviving Sepsis Campaign International Guidelines in 2021 make no mention of VA‐ECMO as a treatment modality for refractive septic shock. One reason for this may be the wide variation in study‐reported survival rates of refractive septic shock patients treated by VA‐ECMO, ranging from 15% to 70%.^2^, ^3^ At our institution, we consider the introduction of VA‐ECMO in cases of refractory septic shock only with cardiogenic shock due to septic cardiomyopathy.

The present case had left ventricular wall hypokinesia indicative of reverse Takotsubo cardiomyopathy, which suggested one of the following three pathological conditions or a combination of several: (1) septic cardiomyopathy, (2) catecholamine cardiomyopathy, and (3) left ventricular overload due to excessive transfusion.^4^, ^5^ It was impossible to clinically differentiate between the three at that point because the patient's hemodynamics was too unstable to reduce the catecholamines and the transfusions. For (2) catecholamine cardiomyopathy and (3) left ventricular overload due to excessive transfusion, there seemed to be a possibility of recovery if the catecholamines could be tapered and the fluids could be drained by introducing ECMO to support circulation. As for (1) septic cardiomyopathy, we usually consider it as an indication for VA‐ECMO. However, this patient presented an immunocompromised state with marked neutropenia, which is one of the exclusion criteria in the ELSO guidelines^1^ probably because it reduces the probability of recovery from sepsis.

The mortality rates of immunosuppressed septic patients in ICU depend on the cause of immunosuppression, which is categorized into three groups: solid tumors including postchemotherapy, hematologic malignancies, and nonmalignant immunosuppression. The mortality rates of patients with sepsis in these groups were reported to be 20%–70%, 30%–80%, and 30%–40%, respectively, with considerable variation among previous studies.^6^, ^7^ Some reports have shown that performance status and organ dysfunction have a greater impact on mortality than the immunodeficiency type.^6^ Neutropenia is also an independent risk factor for hospital mortality of patients with malignancies and sepsis^7^; however, the mortality rate has improved substantially in the last two decades,^8^ and it is not a contraindication for intensive care, except in cases of extremely poor prognosis of the malignancy. Recent cohort studies on septic shock treated with VA‐ECMO included 11–65% immunocompromised patients, although the mortality rate was not reported.^9^

In the present case, the immunodeficiency was caused by chemotherapy for a solid tumor, which has a somewhat higher mortality rate compared with nonmalignant immunosuppression. However, the solid tumor was an invasive mole, whose overall cure rate with chemotherapy is nearly 100%. The patient was relatively young with a good performance status before the sepsis, which also lowered the mortality prediction. She had marked thrombocytopenia and coagulopathy; however, these major risk factors of hemorrhagic complications could be treated with blood transfusions. Despite severe leukopenia on admission, her leukocyte count showed a slight increase with granulocyte‐stimulating factor, which seemed to indicate the possibility of recovery if more time could be provided for infection control by supporting the circulation with VA‐ECMO. Therefore, VA‐ECMO, despite being a controversial method for use in such cases, was administered as there was a good chance of recovery based on those factors, and the patient survived the septic shock. This case demonstrated that VA‐ECMO support may be effective for refractory leukopenic septic shock in certain conditions.

## 4 CONCLUSION

VA‐ECMO for leukopenic septic shock is a viable treatment option for relatively young patients with manageable underlying diseases.

## AUTHOR CONTRIBUTIONS

**Atsumi Hoshino:** Conceptualization; data curation; writing – original draft. **Shingo Ichiba:** Supervision; validation. **Junya Ishikawa:** Data curation. **Yusuke Seino:** Investigation; visualization. **Takuo Yoshida:** Data curation. **Nobuo Sato:** Data curation. **Takeshi Nomura:** Resources; supervision; validation.

## FUNDING INFORMATION

Not applicable.

## CONFLICT OF INTEREST STATEMENT

The authors declare that they have no conflict of interest associated with this manuscript.

## ETHICS STATEMENT

Ethical review and approval were not required for the study in accordance with the local legislation and institutional requirements.

## CONSENT

Written informed consent was obtained from the patient for the publication of this report.

## Data Availability

The raw data supporting the conclusions of this article will be made available by the authors, without undue reservation.

## References

[1] Lorusso R , Shekar K , MacLaren G , et al. ELSO interim guidelines for venoarterial extracorporeal membrane oxygenation in adult cardiac patients [published correction appears in ASAIO J 2022 Jul 1;68(7):e133]. ASAIO J. 2021;67(8):827‐844. doi:10.1097/MAT.0000000000001510 34339398

[2] Sato R , Kuriyama A . Venoarterial extracorporeal membranous oxygenation: treatment option for sepsis‐induced cardiogenic shock? A systematic review. Crit Care Med. 2020;48(8):e722‐e729. doi:10.1097/CCM.0000000000004432 32697514

[3] Ling RR , Ramanathan K , Poon WH , et al. Venoarterial extracorporeal membrane oxygenation as mechanical circulatory support in adult septic shock: a systematic review and meta‐analysis with individual participant data meta‐regression analysis. Crit Care. 2021;25(1):246. doi:10.1186/s13054-021-03668-5 34261492 PMC8278703

[4] L'Heureux M , Sternberg M , Brath L , et al. Sepsis‐induced cardiomyopathy: a comprehensive review. Curr Cardiol Rep. 2020;22(5):35. doi:10.1007/s11886-020-01277-2 32377972 PMC7222131

[5] Awad HH , McNeal AR , Goyal H . Reverse Takotsubo cardiolyopathy: a comprehensive review. Ann Transl Med. 2018;6(23):460. doi:10.21037/atm.2018.11.08 30603648 PMC6312810

[6] Torres VB , Azevedo LC , Silva UV , et al. Sepsis‐associated outcomes in critically ill patients with malignancies. Ann Am Thorac Soc. 2015;12(8):1185‐1192. doi:10.1513/AnnalsATS.201501-046OC 26086679

[7] Tolsma V , Schwebel C , Azoulay E , et al. Sepsis severe or septic shock: outcome according to immune status and immunodeficiency profile. Chest. 2014;146(5):1205‐1213. doi:10.1378/chest.13-2618 25033349

[8] Legrand M , Max A , Peigne V , et al. Survival in neutropenic patients with severe sepsis or septic shock. Crit Care Med. 2012;40(1):43‐49. doi:10.1097/CCM.0b013e31822b50c2 21926615

[9] Bréchot N , Hajage D , Kimmoun A , et al. Venoarterial extracorporeal membrane oxygenation to rescue sepsis‐induced cardiogenic shock: a retrospective, multicentre, international cohort study. Lancet. 2020;396(10250):545‐552. doi:10.1016/S0140-6736(20)30733-9 32828186

